# Evaluation of environmental factors affecting the genetic diversity, genetic structure, and the potential distribution of *Rhododendron aureum* Georgi under changing climate

**DOI:** 10.1002/ece3.7803

**Published:** 2021-08-25

**Authors:** Wei Zhao, Xiaolong Wang, Lin Li, Jiangnan Li, Hang Yin, Ying Zhao, Xia Chen

**Affiliations:** ^1^ National & Local United Engineering Laboratory for Chinese Herbal Medicine Breeding and Cultivation Jilin University Changchun China; ^2^ School of Life Science Jilin University Changchun China; ^3^ Medical Technology Department Qiqihar Medical University Qiqihar China; ^4^ Jilin Provincial Joint Key Laboratory of Changbai Mountain Biocoenosis and Biodiversity Antu China; ^5^ Academy of Sciences of Changbai Mountain Changbaishan China

**Keywords:** climate change, distribution, environmental factors, genetic diversity, genetic structure, *Rhododendron aureum* Georgi

## Abstract

Understanding genetic variation and structure, adaptive genetic variation, and its relationship with environmental factors is of great significance to understand how plants adapt to climate change and design effective conservation and management strategies. The objective of this study was to (I) investigate the genetic diversity and structure by AFLP markers in 36 populations of *R. aureum* from northeast China, (Ⅱ) reveal the relative contribution of geographical and environmental impacts on the distribution and genetic differentiation of *R. aureum,* (Ⅲ) identify outlier loci under selection and evaluate the association between outlier loci and environmental factors, and (Ⅳ) exactly calculate the development trend of population of *R. aureum*, as it is confronted with severe climate change and to provide information for designing effective conservation and management strategies. We found high genetic variation (*I* = 0.584) and differentiation among populations (*Φ_ST_* = 0.703) and moderate levels of genetic diversity within populations of *R. aureum*. A significant relationship between genetic distance and environmental distance was identified, which suggested that the differentiation of different populations was caused by environmental factors. Using BayeScan and Dfdist, 42 outlier loci are identified and most of the outlier loci are associated with climate or relief factors, suggesting that these loci are linked to genes that are involved in the adaptability of *R. aureum* to the environment. Species distribution models (SDMs) showed that climate warming will cause a significant reduction in suitable areas for *R. aureum*, especially under the RCP 85 scenario. Our results help to understand the potential response of *R. aureum* to climatic changes and provide new perspectives for *R. aureum* resource management and conservation strategies.

## Graphical abstract

Using AFLP markers, landscape genetic, and species distribution modeling analysis together, we are able to identify many environmental factors that have influenced the genetic diversity and genetic structure, and we can predict the potential distribution area of *R. aureum*. Our analyses revealed high genetic variation and differentiation among populations and moderate levels of genetic diversity within populations of *R. aureum*. A significant correlation between genetic distance and environmental distance was identified, which suggested that environmental factors were the primary cause of the population differentiation. A significant correlation between genetic distance and environmental distance was identified, which suggested that environmental factors were the primary cause of the population differentiation.

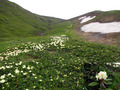

## 1 INTRODUCTION

Genetic diversity is the basic requirement for species to survive long term and to adapt to environmental changes on an evolutionary time scale (Falk et al., 2001; Frankham, 2005). Genetic structure is important as it can provide insights into the history of a population, and the current levels and distribution of genetic variation can influence the future success of populations (Erickson et al., 2004). Under any combination of natural selection and random genetic drift, populations separated by geographic distance may diverge due to reduced gene flow and population connectivity (isolation by geographical distance—IBD) (Nosil & Rundle, 2012). Population divergence may still occur when reproductive isolation evolves between neighboring populations as a result of ecologically based divergent selection in different environments (isolation by environment—IBE) (Wang & Bradburd, 2014). Global climate change has become one of the major threats to biodiversity (Davis & Shaw, 2001; Parmesan, 2006). Species may respond to global climate change by local adaptation (Margaret et al., 2005; Parmesan, 2006), individual migration (Breshears et al., 2008; Lenoir et al., 2008), range reduction (Thuiller et al., 2005), or a combination of these (Margaret et al., 2005). Local adaptation has been found to be a conventional way of responding to climate change in various plant species (Coop et al., 2010; Gonzalez‐Martinez et al., 2006; Hancock et al., 2011; Savolainen et al., 2007). Furthermore, predicted climate warming will have a dramatic impact on mountain ecosystems (Kohler et al., 2010; Rangwala & Miller, 2012), especially for alpine plant communities (Gottfried et al., 2012; Steinbauer et al., 2018). It is therefore crucial to uncover the genetic basis of local adaptations governed by natural selection, which is particularly important for understanding how plants adapt to their environment and respond to climate change.

Alpine tundra is a unique mountain ecosystem, where plants live in harsh environment and are sensitive to climate change. The alpine environment is a local variable as small changes in altitude can lead to large changes in temperature, humidity, exposure, and other types of changes (Byars et al., 2007; Hovenden & Jkvander, 2004). With the global climate changing, in some alpine areas, the increase in air temperature was more than twice as great as the increase in global mean air temperature during the 20th century (Bohm et al., 2001). Plant species are particularly vulnerable under the climate‐changing environment in the alpine. Thus, plants from tundra ecosystems provide a very interesting context to research the genetic variation related to local adaptation and the impact of climate change in plant local adaptation.

**FIGURE 1:**
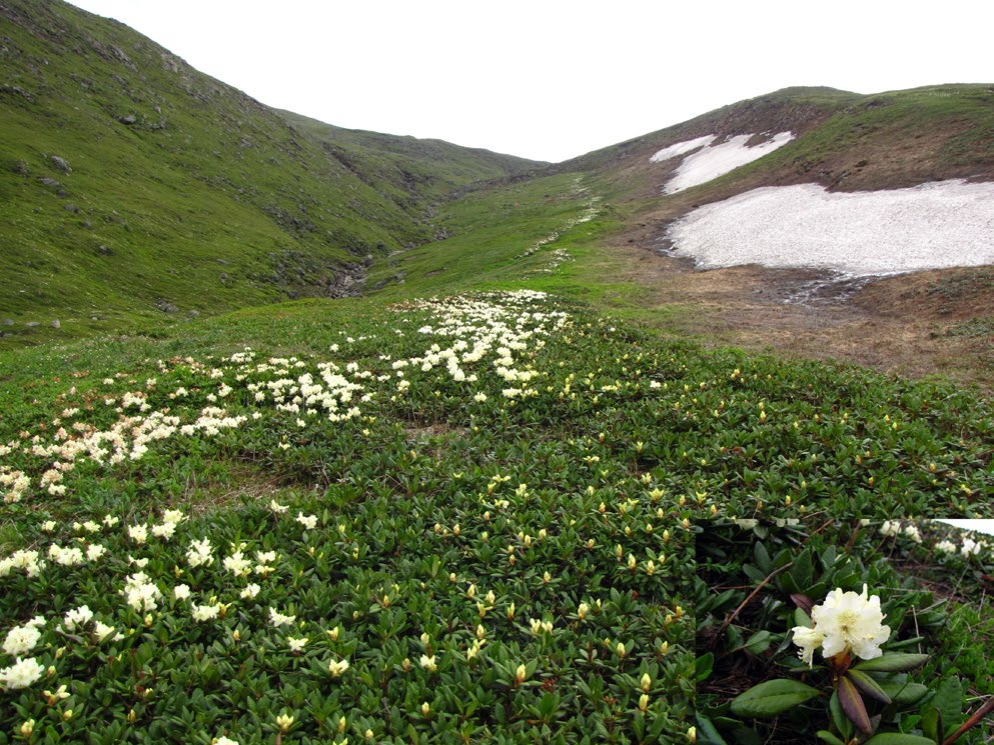
*Rhododendron aureum* Georgi

*Rhododendron aureum* Georgi (syn. *Rh. Chrysanthum* Pall.) (Figure 1), the target plant species in this study, is a perennial evergreen creeping shrub with a large number of branched stems inhabiting alpine regions of Korea, Mongolia, China, Japan, and Russia (Fang et al., 2005). This plant can grow up to 1 m in height and blooms from June to July in Korea with pale yellow flowers. It has been shown to always occupy the snowmelt gradient and especially to dominate in early exposed places (Kudo, 1992). In China, it grows mainly in the alpine tundra and the *Betula ermanii* population belts of Changbai Mountain, ranging from 1,000 to 2,506 m a.s.l. (Kudo, 1993). The *R. aureum* is one of the constructive and dominant species in the alpine tundra ecosystem, and it plays an important role in maintaining the ecological balance and preventing and controlling soil erosion. Understanding the contemporary and historical ecological (climatic, geographical) factors shaping the genetic variation and genetic structure of *R. aureum* is of great significance for studying the effects of climate change in local adaptation.

In this study, we adopted AFLP markers for characterizing the adaptive loci under selection using BayeScan and Dfdist and employed multiple linear regression (MLR) to detect potential adaptive loci that are under selection from existing environmental factors, and used species distribution models (SDMs) to predict the potential distribution of *R. aureum* during the last glacial maximum (LGM) and the future. The objective of this study was to (a) investigate the genetic variation and genetic structure of *R. aureum*; (b) reveal the relative contribution of geographical and environmental impacts on the distribution and genetic differentiation of *R. aureum,* (c) identify outlier loci under selection and assess the association between outlier loci and climate, and (d) calculate development trend of population of *R. aureum*, as it is confronted with severe climate change and to provide information for designing effective conservation and management strategies.

## 2 MATERIALS AND METHODS

### 2.1 Study site

Changbai Mountain is generally recognized as the highest peak in northeast China, with obvious mountain climate characteristics. This environment is a local variable as small changes in altitude can lead to large changes in temperature, humidity, exposure, and other types of changes (Yang & Wu, 1998). The varied topography, weather, soil, and other natural conditions have created rich biodiversity and vertical zonal distribution of vegetation on Changbai Mountain, which has more than 2,277 species of plants and a notable richness of endemic species.

### 2.2 Genetic analyses of *R. aureum*

**FIGURE 2:**
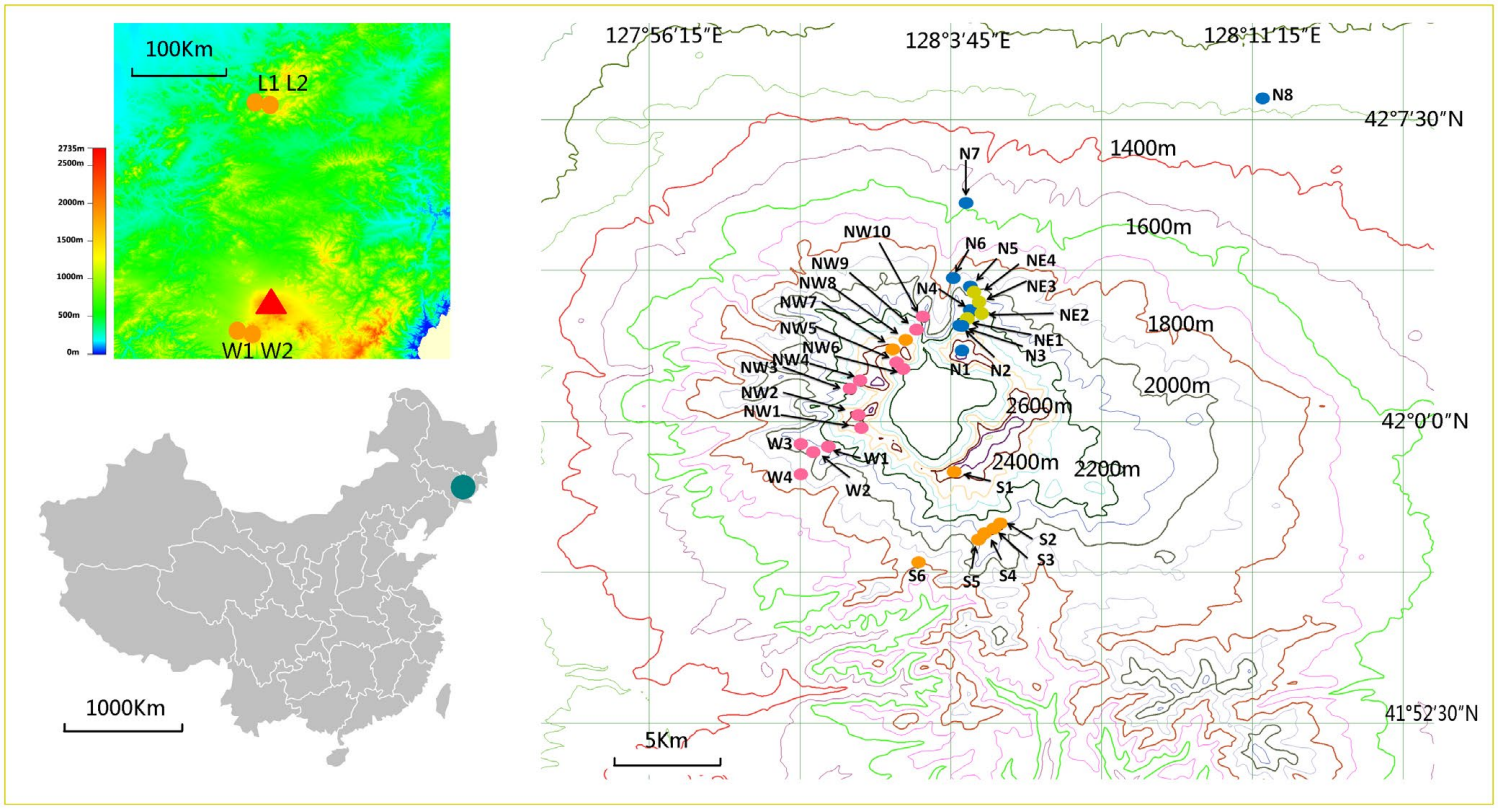
Distribution of 36 *R. aureum* *populations* sampled from China. We extracted the elevational information from DIVA‐GIS Free Spatial Data (http://www.diva‐gis.org/Data) and created the map by the program Global Mapper version 13.00 (http://www.bluemarblegeo.com/products/global‐mapper.php) and DIVA‐GIS version 7.5.0

From 2012 to 2014, fresh leaves were collected from 461 individuals belonging to 36 nature populations of *R. aureum* along an environmental transect, which altitude ranges from 1,200 m to 2,600 m a.s.l., annual mean temperature ranges from 0.1°C to −6.6°C, and annual precipitation ranges from 761 mm to 1,096 mm (Table 1, Figure D). These populations were scattered along peaks of Changbai Mountain (32 populations), Laobai Mountain (2 populations), and Wangtian'e Mountain (2 populations) (Figure 2). In each of the populations, a random sample from 7 to 17 individuals was obtained. In each locality, individual samples were taken from plants separated by at least 5 m (in order to avoid sampling clones) and dried directly in silica gel for transport back to Jilin University for subsequent DNA extraction.

**TABLE 1:** Summary of AFLP variation for 36 populations of *R. aureum*

Pop	GPS coordinates (N/E)		Altitude (m)	*N*	PPL	*I*	*H*
N1	42.0292	128.0664	2,600	12	41.871	0.228	0.154
N2	42.04018	128.0678	2,300	8	58.797	0.329	0.224
N3	42.04202	128.0686	2,200	14	31.403	0.153	0.100
N4	42.04575	128.0706	2,100	14	22.272	0.123	0.083
N5	42.05552	128.0699	2,000	10	37.862	0.208	0.142
N6	42.05935	128.063	1,800	11	33.185	0.188	0.128
N7	42.09014	128.0687	1,580	14	2.450	0.014	0.010
N8	42.13372	128.191	1,200	14	18.708	0.103	0.070
NW1	41.99727	128.025	2,462	14	52.116	0.293	0.200
NW2	42.0021	128.0249	2,590	14	56.570	0.315	0.214
NW3	42.01297	128.0212	2,161	9	30.958	0.166	0.112
NW4	42.0164	128.0252	2,501	14	33.185	0.179	0.121
NW5	42.02153	128.0425	2,198	7	32.071	0.176	0.119
NW6	42.02443	128.0402	2,341	10	55.011	0.302	0.205
NW7	42.03347	128.0433	2,431	14	41.425	0.212	0.140
NW8	42.03802	128.0485	2,315	14	38.307	0.193	0.128
NW9	42.02957	128.0385	2,500	7	39.421	0.225	0.153
NW10	42.043	128.0515	2,122	14	36.526	0.189	0.126
W1	41.99007	128.0001	2,223	14	35.857	0.196	0.133
W2	41.98912	128.0119	2,138	14	32.517	0.163	0.109
W3	41.98713	128.0049	2,014	14	30.067	0.154	0.102
W4	41.97795	128.0006	1,872	14	33.408	0.171	0.114
S1	41.97879	128.0636	2,535	14	48.998	0.257	0.172
S2	41.95675	128.0826	2,099	14	34.744	0.179	0.12
S3	41.95501	128.0794	2,083	14	37.639	0.198	0.133
S4	41.95376	128.0762	2,030	14	50.78	0.278	0.188
S5	41.95178	128.0752	1,945	14	35.412	0.177	0.118
S6	41.94115	128.0488	1,814	14	30.735	0.181	0.125
NE1	42.0542	128.0714	2,000	T	33.63	0.189	0.129
NE2	42.04927	128.0738	2,100	9	29.176	0.172	0.118
NE3	42.04517	128.0749	2,200	14	29.844	0.159	0.107
NE4	42.04015	128.0681	2,300	14	29.399	0.156	0.105
L1	44.10465	128.0431	1,698	14	39.866	0.218	0.147
L2	44.10325	128.0426	1,701	14	34.967	0.185	0.125
WTE1	41.72863	127.902	2,048	14	25.612	0.124	0.083
WTE2	41.72845	127.9011	2,044	14	26.949	0.115	0.074
Total	–	–	–	461	99.777	0.584	0.402

Abbreviations: *H*, Nei's (1973) gene diversity; *I*, Shannon's information index; *N*, population size; PPL, the percentage of polymorphic loci.

The total DNA was extracted from the dried leaves by using a Plant Genomic DNA Kit (Biotech Beijing Co. Ltd., Beijing, China). The DNA samples were diluted to 10 ng/μl and stored at −20°C until further analysis. AFLP marker was carried out according to the method of Vos et al. (1995) with the following little modifications: The digestion–ligation reaction was performed in a 10 μl containing 1 μl of 10 × T4 ligation buffer, 0.2 mM of ATP, 50 ng DNA, 1 U of T4 DNA ligase (Fermentas, Shenzhen, China), 1 U of *Eco*RI (Fermentas, Shenzhen, China), 1 U of *Mse*I (New England Biolabs, Beijing LTD), 1.0 μM of *MseI*‐Adapter, and 0.1 μM of *Eco*RI‐Adapter and double‐distilled water. The reaction was incubated at 37°C for 8 hr, 16°C for 4 hr, inactivated at 65°C for 10 min, and stored at 4°C. Pre‐amplification was performed in a 25 μl containing 2.5 μl of 10× PCR buffer and 0.5 U of Taq polymerase (Transgen Biotech Beijing Co. Ltd., China), 2.5 μl of diluted digestion–ligation product, 0.2 μM of dNTPs, and 0.3 μM each of primers with a single selective nucleotide. The pre‐amplification conditions were as follows: predenatured at 94°C for 5 min and 30 cycles of 94°C for 30 s, 56°C for 60 s, and 72°C for 80 s, with a final extension for 10 min at 72°C. The pre‐amplification products were diluted 1 to 40 (v/v) with ddH_2_O. The selective amplification was essentially the same as that for pre‐amplification except that 2 μl diluted pre‐amplification product was used as a template, and 2 μM *Eco*RI and *Mse*I selective primers were used. 10 pairs of primers were selected for selective amplification (Table A). The selective amplification reaction had two cycle sets: predenatured at 94°C for 5 min, 13 cycles of 94°C for 30 s, 65°C (which was lowered 0.7°C at each cycle) for 30 s and 72°C for 60 s, followed by 18 cycles of 94°C for 30 s, 56°C for 30 s and 72°C for 80 s. After selective amplification, the products were added 25 μl of formamide loading buffer (98% deionized formamide, 10 mM EDTA, 0.1% bromophenol blue, and 0.1% xylene cyanol). Then, the products were denatured at 95°C for 5 min and quickly cooled on ice, and separated on 6% denaturing polyacrylamide gel in 1× TBE buffer at 70 W for 4.5 hr. Gels were stained according to the silver staining method (Bassam et al., 1991).

The AFLP bands were scored as present (1) or absent (0), and the AFLP band data were transferred to a binary (1/0) data matrix for further analysis. Shannon's information index (*I*) (Lewontin, 1972), percentage of polymorphic loci (*PPL*), and genetic distance were estimated using the POPGENE v1.31 (Yeh et al., 1997). Total genetic diversity (*H_T_*) and mean genetic diversity within populations (*H_S_*) were calculated using Nei's (Nei, 1973) genetic diversity statistics. Population genetic structure was further assessed using model‐based Bayesian assignment as implemented in STRUCTURE 2.3.4 software (Pritchard et al., 2000). Clustering of individuals was constructed without using the geographical origin of the samples as an informative prior. Analyses were based on an admixture ancestry model with correlated allele frequencies for a range of *K* genetic clusters from 1 to 36, with 20 replicates per K. The analyses were performed with a burn‐in period and a run length of the Monte Carlo Markov chain (MCMC), of 20,000 and 200,000 iterations, respectively. The most likely number of genetic clusters (*K*) was estimated by the Δ*K* statistic (Evanno et al., 2005), using Structure Harvester (Earl & Vonholdt, 2012). Then, CLUMPP 1.1.2 (Jakobsson & Rosenberg, 2007) was used to align the 20 runs of the most representative *K* value and to compute the pairwise symmetric similarity coefficients (*G*) between pairs of runs, the average pairwise similarity (*H*) for the 20 replicates, and the average probability of belonging to each cluster (*Q*). For *K* = 4, the full search method with 1,000 replicates was used. A hierarchical analysis of molecular variance (AMOVA) was used to determine genetic differentiation (*F_ST_* s within and among the groups. A nested AMOVA takes into consideration the four main genetic groups resulting from the Bayesian clustering with STRUCTURE (*K* = 4) using ARLEQUIN version 3.5.1.2 (Excoffier & Lischer, 2010).

Simulations were conducted with a mean *F_ST_* equal to the trimmed mean *F_ST_*, which was calculated by excluding 30% of the most extreme *F_ST_* values observed in the empirical dataset. We analyzed the distributions of the *F_ST_* values over all loci to null hypothesis of neutral evolution. Loci with a high or low *F_ST_* value were taken as potentially under selection. In the study, we simulated the neutral distribution of *F_ST_* with 10,000 iterations at the 99.9% confidence intervals.

### 2.3 Environmental data and correlation analyses

To characterize environmental differences, BIOCLIM variables were obtained for each of the 36 sites by extrapolating climate data to the GPS coordinates for each population using DIVA‐GIS software (Hijmans et al., 2001, 2005). The BIOCLIM dataset includes 19 variables that describe monthly temperature and precipitation patterns for a spatial resolution of 1 km^2^ (http://www.worldclim.org/). The sampled area spanned all the known *R. aureum* range in China, which occurs primarily in the alpine, along an annual mean precipitation gradient from 761 to 1,096 mm and annual mean temperature gradient from −6.6°C to 0.1°C. Elevation, slope, and topographic index were derived from a 90 m digital elevation model. The dataset is provided by International Scientific & Technical Data Mirror Site, Computer Network Information Center, Chinese Academy of Sciences (http://www.gscloud.cn). A principal component analysis (PCA) was applied to these environmental variables to examine possible correlations between ecoclimatic variables and elevation and remove redundant variables (i.e., variables that were correlated at |*r*| > .8 and which were logically related). We first identified variables correlated with each retained axis, creating groups of variables. Within each group, we kept only one (or two) variables considered to be the most pertinent in terms of local adaptation in plants. Finally, eleven factors (Bio 1, annual mean temperature; Bio 2, mean diurnal range; Bio 3, Isothermality; Bio 4, temperature seasonality; Bio 5, max temperature of warmest month; Bio 9, mean temperature of driest quarter; Bio 16, precipitation of wettest quarter; Bio 17, precipitation of driest quarter; slp, slope; asp, aspect; tpi, topographic position index) were chosen as representative of environment factors.

Prior to subsequent analyses, environment data were log_10_(*x* + 1) transformed to improve normality and reduce heteroscedasticity. Dissimilarity matrices of Euclidean distances were calculated among normalized environment variables using R package. A matrix of geographic distances among sites was generated from GPS coordinates with the AFLP data in R software (Ehrich, 2006) and also log_10_(*x* + 1) transformed. The genetic distance matrices of *R. aureum* were calculated with PopGene (Yeh et al., 1997) using the Jaccard dissimilarity method of the scored bands and were tested against the Euclidean distance of the environment variables while controlling the geographic distance matrix. The Mantel test was performed using the default preset values and parameters (999 permutation steps) according to the software manual using R program “vegan” package. Multiple matrix regression with randomization (MMRR) is a novel and robust approach for estimating the independent effects of potential factors, and the analysis was implemented with 10,000 permutations in R with the MMRR function script (Goslee & Urban, 2007; Wang, 2013; Wu et al., 2015).

Then, to detect relationships between allele frequencies and environmental variables, we applied multiple linear regression (MLR) (Zulliger et al., 2013) in R v.3.3.3 to determine potential adaptive loci that are under selection from existing environmental factors. For outlier loci identified by both Dfdist and BayeScan, we estimated their population pairwise frequencies of AFLP alleles at the 36 sites. We selected values of the eleven selected environmental factors from each population. We then regressed the allele frequencies of the retained outlier loci (dependent variables) on the selected environmental variables (explanatory variables; standardized) using the MLR model. Potential adaptive loci were identified as $R_{\text{adj}}^{2}$ > 0.5. Univariate regressions were then applied to each variable individually to estimate its significance.

### 2.4 Species distribution model (SDM)

We used Maxent 3.3.3k to predict distribution changes for *R. aureum* as a result of climate changing. Maxent is a program for maximum entropy modeling of the geographical distributions of species; it combines presence‐only data with ecological‐climatic layers to predict suitable areas (Phillips et al., 2006; Phillips & Dudik, 2008). For current distribution, we downscaled climate grids for the periods 1970–2000. In addition to sample locations in this study, we also collected the distribution records of *R. aureum* from the Chinese Virtual Herbarium (http://www.cvh.ac.cn/). After removing duplicate records, it remained a total of 42 records of *R. aureum* (Table A) that were used to establish the distribution model by using 19 bioclimatic data layers from the WorldClim database. Most of the default parameters of Maxent were applied to conduct SDM, except the following user‐selected parameters: application of random seed and random test percentage of 70%, replicates of 10 and bootstrap as the replicated run type. The logistic output of Maxent consists of a grid map with each cell having an index of suitability between 0 and 1. Low values show that the conditions are unsuitable for the species to live, whereas high values show that the conditions are more suitable. Model predictions were visualized using DIVA‐GIS (Hijmans et al., 2001).

To obtain the distribution of *R. aureum* at the last glacial maximum, we projected correlation between current species–climate and the LGM using the Community Climate System Model (CCSM4) scaled down to a 2.5‐arcmin resolution. We used the Hadley Global Environment Model 2 (HadGEM2‐ES) as a general circulation model under two climate scenarios (IPCC‐CMIP5 RCP 26/85) to ensure the accuracy of assessment. The RCP 85 scenario represents a higher predicted greenhouse gas emission than RCP 26.

## 3 RESULTS

### 3.1 Patterns of AFLP variation and population structure

The ten AFLP primer combinations generated 449 unambiguously scorable bands ranging from 1,500 to 100 bp in 461 individuals from 36 natural populations. Of these fragments, 99.777% (448) were polymorphic. The overall Shannon's information index (*I*) and Nei's genetic diversity (*H*) were 0.584 and 0.402, respectively. *R. aureum* showed high genetic diversity at the species level. *I* ranged from 0.014 to 0.329, and *H* ranged from 0.01 to 0.228 within population (Table 1). The genetic diversity of most populations is at moderate level, but there is still a low genetic diversity of some populations, such as population N7.

**FIGURE 3:**
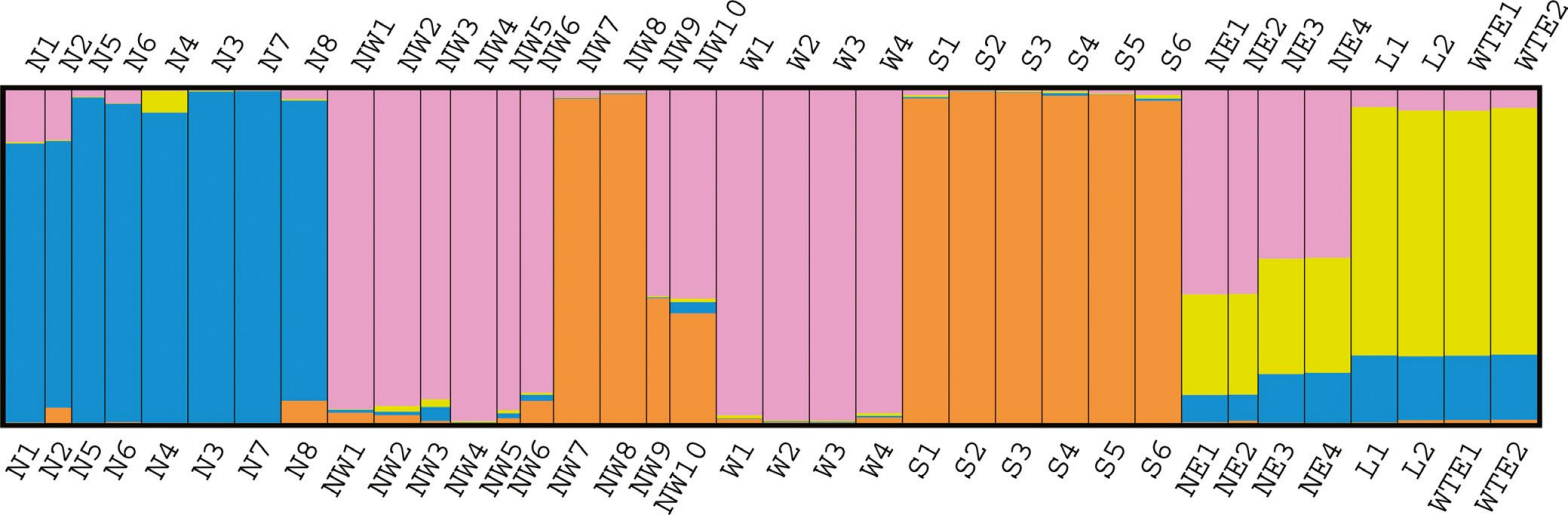
Genetic structure of 36 *R. aureum* populations inferred from AFLP data using the STRUCTURE result at *K* = 4

Bayesian clustering analyses performed with the software STRUCTURE indicated that the most informative representation of overall genetic structure was achieved for *K* = 4 (Appendix C), where *K* is the number of subpopulations. In all northern slope populations, N1–N8 formed the group 1 and showed some degree of genetic admixture from other populations. The northwestern populations NW1‐NW6 and NW9, NW10, and the western populations W1‐W4 formed the group 2, in which NW9 and NW10 had obvious genetic admixture. The southern slope populations (S1–S6) and NW7 and NW8 populations on the U‐shaped valley of the northern slope formed the group 3. Populations L1, L2, WTE1, and WTE2 formed the group 4, and their individuals had a high level of admixture (Figure 3).

An AMOVA (Table 2) attributed 68.87% of the overall genetic variation to the among‐population component. A nested AMOVA that considered the four main genetic groups based on the Bayesian clustering analysis with the software STRUCTURE (*K* = 4) attributed 16.8% of the global variation to differences between the 4 groups, 52.14% to among populations within groups and 31.06% to within populations (*Φ_ST_* = 0.703; both at *p* < .0001).

**TABLE 2:** AMOVAs for AFLP variation surveyed in a total of 36 populations of *R. aureum*

Structure analyzed and source of variation	*df*	Sum of squares	Variance components	Variation (%)	*Φ_ST_* (95% CI)	*Φ_CT_* (95% CI)
(a) Global analysis					0.689	
Among populations	35	27,453.15	59.21	68.87		
Within populations	425	11,373.28	26.76	31.13		
(b) Grouping following STRUCTURE clustering (*K* = 4)					0.703	0.18507
Among groups	3	7,086.82	15.03	16.8		
Among populations within groups	32	19,930.62	46.65	52.14		
Within populations	425	11,809.75	27.79	31.06		

### 3.2 Correlations between genetic variation and environmental versus geographical factors

The Mantel test showed a significant correlation between genetic distance and environmental distance (*r* = .4871, *p* = .001), but between genetic distance and geographical distance were a nonsignificant correlation (*r* = .0971, *p* = .028). When geographical factors were controlled, a partial Mantel test also showed isolation by environmental distance (*r* = .4797, *p* = .001). Whereas environmental factors were controlled, we could not detect significant correlations between genetic differentiation and geographical distance (*r* = .0118, *p* = .378). The MMRR analysis indicated that the environment factors had large regression coefficient, whereas the effects of geographic distance were not significant (geographic distance: *β* = 0.0094, *p* = .1075; environment distance: *β* = 0.2372, *p* = .0001; Table 3).

**TABLE 3:** Results of the Mantel test, partial Mantel test, and MMRR analyzing the correlation between geographical distances, environmental distances, and Nei's genetic distance based on AFLP data

	Mantel test		Partial Mantel test		MMRR	
	*r*	*p* value	*r*	*p* value	*β*	*p* value
Gen.Geo	.0971	.028	.0118	.378	0.0094	.1075
Gen.Env	.**4871**	.**001**	.**4797**	.**001**	**0.2372**	.**0001**

Note

Regular letters refer to nonsignificant results and bold letters refer to significant correlations.

Abbreviations: Env, environmental distance; Gen, genetic distance; Geo, geographical distance.

### 3.3 Outlier analyses and MLR analysis

**FIGURE 4:**
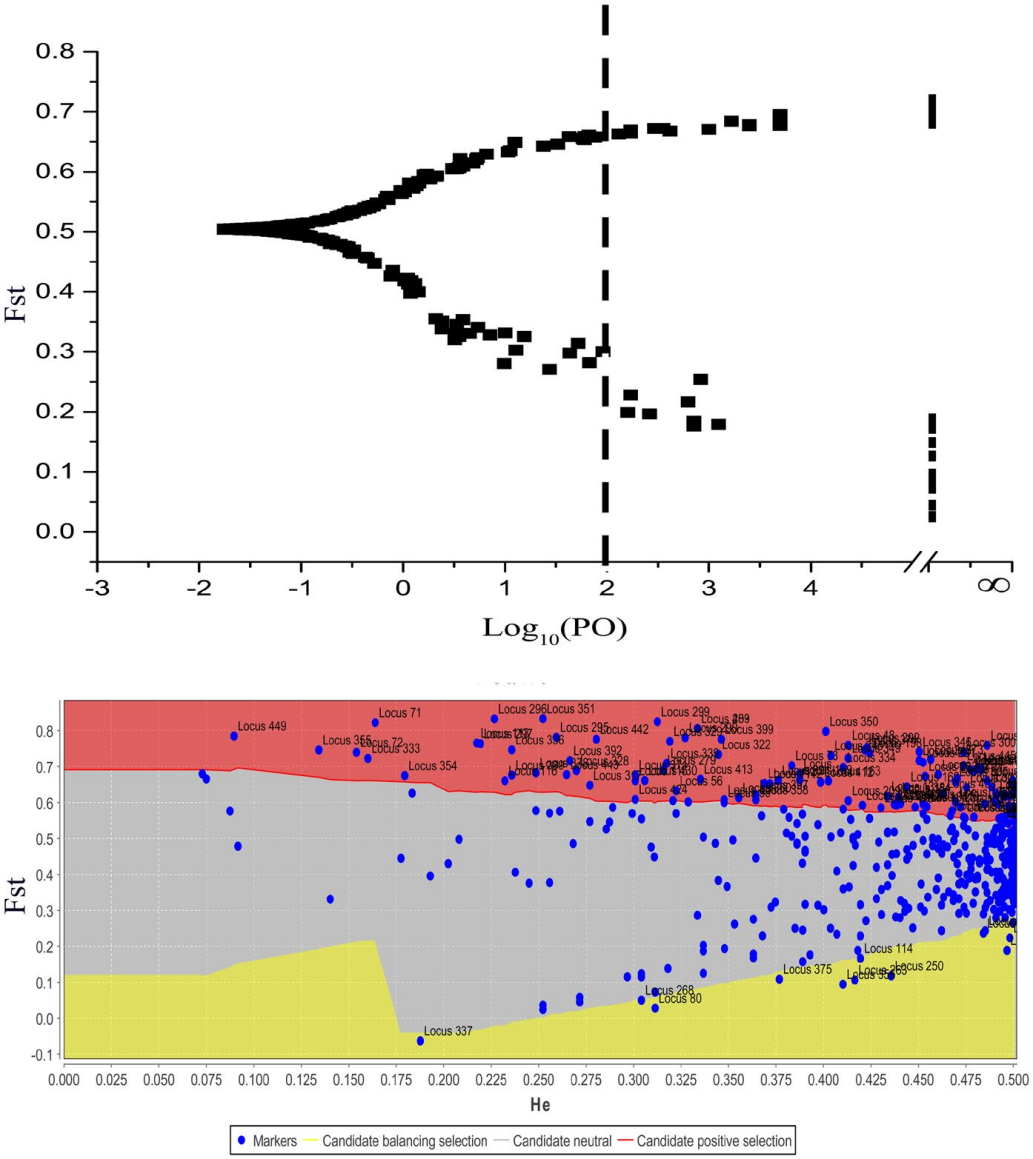
Outlier loci identified by BayeScan and Dfdist. (a) Plot of *F_ST_* values and log_10_PO for 461 loci identified using BayeScan. Lines log_10_PO = 2 indicate “decisive” evidence for selection corresponding to a posterior probability of 0.99. Solid black dots greater than log_10_PO 2 represented outlier loci. (b) Outlier detection was performed with Dfdist. Plot of *F_ST_* values of 461 loci in *R. aureum* populations was against heterozygosity

BayeScan determined 71 loci as outliers with a log_10_PO above 2, which is a threshold for adequate evidence for accepting a model under selection, corresponding to a posterior probability greater than 0.99 (Figure 4a). Using the Dfdist, we identified 126 adaptive loci at the 99.5% confidence intervals (Figure 4b). 42 outlier loci were identified using two complementary analyses. The extremely strict significance criteria in the two approaches also assured the robustness of 42 outlier loci. Lastly, 21 potential loci under selection were verified by the MLR analysis with $R_{\text{adj}}^{2}$ > 0.5 (Table 4). When we ran linear regressions using each environmental variable individually, all these eleven environmental variables were associated with the potential loci under selection and 30 loci were significantly (*p* < .05) associated with at least one of the eleven selected environmental variables.

**TABLE 4:** Results of outliers and environmental association analyses on AFLP loci of *R. aureum*

Outlier ID	Environmental variables $R_{\text{adj}}^{2}$	Significant environmental variables	Outlier ID	Environmental variables $R_{\text{adj}}^{2}$	Significant environmental variables
**L48**	**0.52**	BIO3,BIO9	L348	0.43	slp
**L73**	**0.64**	BIO1, BIO3, BIO9	L349	0.39	–
L198	0.45	BIO2, slp	**L350**	**0.58**	BIO3
**L283**	**0.52**	BIO2	**L352**	**0.53**	asp
**L287**	**0.73**	BIO2, BIO16, BIO17, tpi	L373	0.30	–
**L289**	**0.61**	BIO1, BIO3	L374	0.36	–
**L290**	**0.54**	BIO1, BIO3	**L399**	**0.64**	BIO1, BIO5, asp
**L291**	**0.50**	BIO1, BIO3	**L403**	**0.63**	BIO1, BIO5
**L292**	**0.51**	BIO3	**L404**	**0.55**	BIO3, asp
**L293**	**0.66**	BIO3	L405	0.49	BIO3, BIO17
**L294**	**0.57**	BIO3	L445	0.28	–
**L298**	**0.58**	BIO3,tpi	L53	0.34	BIO4, BIO9
**L299**	**0.65**	BIO2, BIO3, tpi	L59	0.42	–
L300	0.33	tpi	L80	0.40	BIO1
**L327**	**0.64**	BIO1, BIO9, tpi, slp	L250	0.42	BIO3, BIO16, BIO17
L332	0.33	–	L263	0.19	–
**L334**	**0.59**	tpi	L268	0.28	–
**L335**	**0.58**	BIO2, tpi, slp	**L304**	**0.62**	BIO2, asp
L344	0.33	–	L337	0.18	–
L345	0.41	–	L356	0.42	BIO17
L346	0.47	BIO4, BIO16, asp	L375	0.28	–

Note

Bold, $R_{\text{adj}}^{2}$ > 0.5.

### 3.4 LGM, present and future distribution of *R. aureum*

**FIGURE 5:**
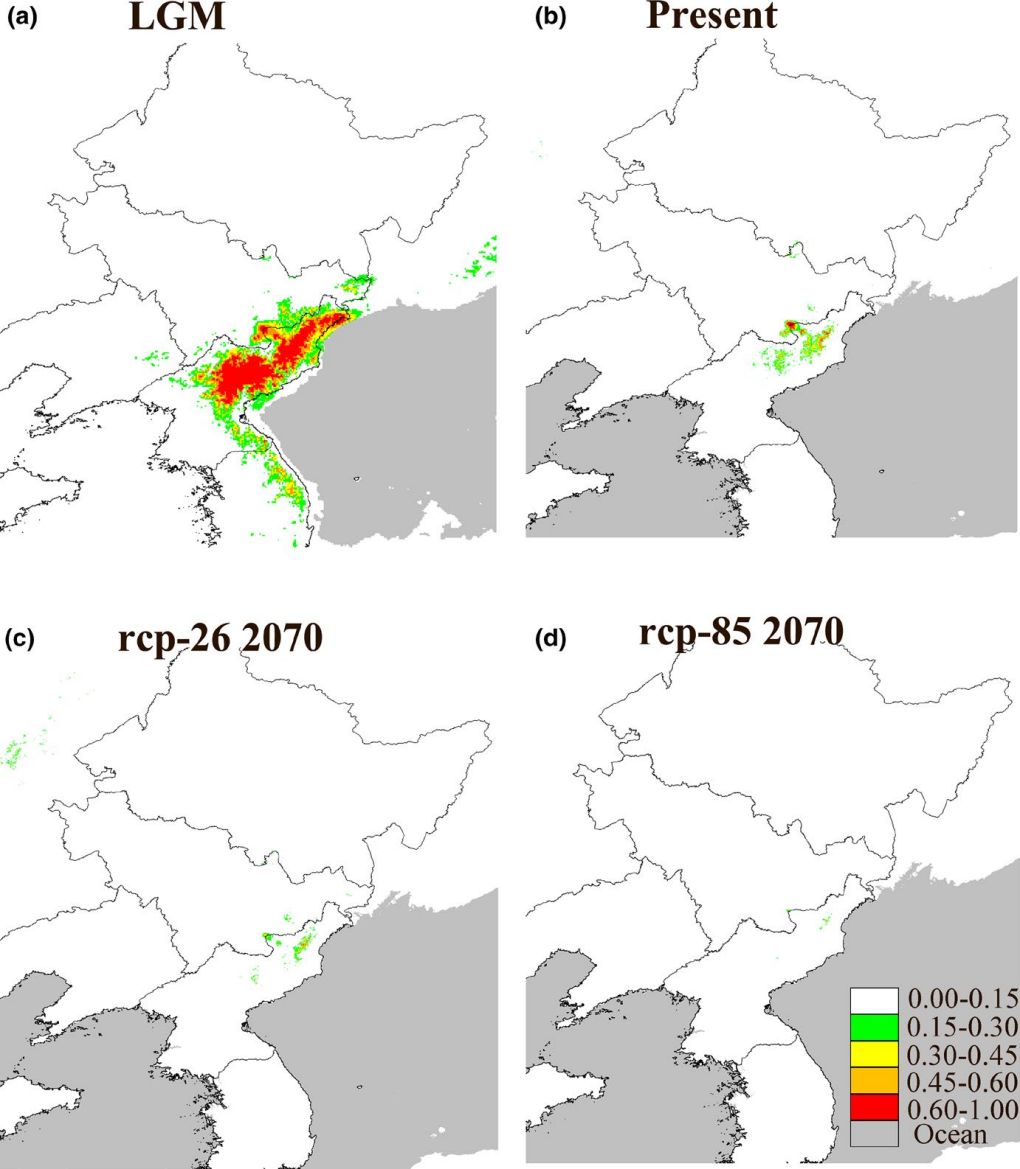
Potential distributions as the probability of occurrence for *R. aureum*. Suitability values indicate logistic probabilities ranging from 0 to 1, with increasingly darker shades of red with increasing habitat suitability. (a) Last glacial maximum (LGM) scenario, (b) present scenario, (c) RCP 26 scenario, and (d) RCP 85 scenario (MAXENT v3.3.3k & Adobe Photoshop CS3)

The average training AUC for ten replicate runs is 0.981, and the standard deviation is 0.035 which indicated an excellent predictive model performance. Minimum training presence logistic threshold was 0.15. The predicted distribution of *R. aureum* (Figure 5b) is consistent with the present distribution records including Changbai Mountain, Wangtian'e Mountain, Laobai Mountain, and North Korea, showing that the distribution of *R. aureum* is conditioned by environmental factors. The distribution of the LGM based on CCSM4 (Figure 5a) was substantially different from the present. The assessed distribution of *R. aureum* during the LGM was expanded to Korean Autonomous Prefecture of Yanbian and northern mountain of Korean peninsula. The predicted future and present distribution of *R. aureum* was considerably different in geographical range (Figure 5c,d). The major difference was that the predicted future suitable habitats showed a significant lessening in comparison with the present one. Only the peak of Changbai Mountain and some area in the North Korea would suitable for *R. aureum* under the rcp85 scenario. Loss of suitable habitats because of the climate changing indicated a drastically range contraction.

## 4 DISCUSSION

### 4.1 Current genetic diversity and differentiation of *R. aureum*

*R. aureum* exhibited a higher level of genetic diversity at the species level (*I* = 0.584, *H* = 0.402), and the high level of genetic diversity was also in accordance with studies on five *Rhododendron* species in Qinling Mountain in China, and Shannon's information index (*I*) differed from 0.4286 to 0.6921, while Nei's gene diversity (*H*) varied from 0.2711 to 0.4989 (Zhao et al., 2012). For 22 populations of 5 wild *Rhododendron* species in Qinling Mountain, as for *R. aureum* more populations were examined, which can fully show local‐level genetic diversity of *R. aureum* in Changbai Mountain. High genetic diversity was also observed in *R. aureum* populations by other molecular markers, such as RAPD, ISSR, and SSR (Liu et al., 2012; Polezhaeva et al., 2021). Different molecular marker methods showed the same results, which fully proved that *R. aureum* has high genetic diversity.

Plant species with wide altitudinal ranges encounter different environmental conditions across the elevation gradient, which may lead to genetic variation as well as phenotypic variation among populations (Forsman, 2014; Nicotra et al., 2015; Ohsawa & Ide, 2008). *R. aureum* is a long‐lived, perennial, evergreen, dwarf shrub which altitude range from 1,000 m to 2,600 m in alpine regions. Along elevation gradients of alpine area, large changes in environmental factors, such as temperature, precipitation (Figure D), solar radiation, and wind, occur over short distances, resulting from obvious changes in the selection pressures of *R. aureum* individuals.

Heterogeneous habitats strengthen disruptive selection to increase variation, and divergent selection pressures promote the evolution of traits adapted to their local environment (Freeland, 2005). Divergent selection can promote genetic differentiation by reducing gene flow among sites with contrasting ecological conditions (Forester et al., 2016). Results also showed that the genetic variability was even greater among populations (68.87%) but smaller within populations (31.13%), and there are high levels of differentiation among populations (*Φ_ST_* = 0.689). Meanwhile, the high population differentiation could possibly accelerate local adaptation. Local adaptation and directional selection should have locus‐specific effects of reducing genetic diversity within populations and increasing differentiation between populations (Magdy et al., 2016). Furthermore, long‐lived perennial species with mixed breeding systems usually have relatively high genetic diversity (Nybom & Bartish, 2000). In the long‐term evolutionary process, the high genetic variation held by *R. aureum* may have provided abundant genotypes for its adaptation to changing climatic conditions. Genetic divergence between populations is shaped by a combination of drift, migration, and selection, yielding patterns of isolation by distance (IBD) and isolation by environment (IBE) (Weber et al., 2017). Some researches on population genetic structure discovered that IBD plays a more important role in intraspecific genetic differentiation than IBE (Mosca et al., 2014); however, IBE was implied to have a stronger effect than IBD on genetic structure in other plant taxa (Gray et al., 2014). A stronger effect of IBE versus IBD was found for the genetic differentiation of *R. aureum*. A Mantel test, partial Mantel test, and MMRR analysis all supported the effect of isolation by environmental distance. In the genetic structure analysis, the fact that some geographically close populations are separated by larger genetic divergence than expected also proved the IBD is not the major driver of population divergence of *R. aureum*. The prominence of IBE suggests factors related to the environment play a greater role in divergence of *R. aureum* populations than geographical isolation. *R. aureum* lives in diversified habitats across its distribution region, and ecological landscape heterogeneity may influence gene flow and connectivity among populations that are adapted to different environments. Possible mechanisms responsible for IBE are selection pressures from climate and relief factors.

### 4.2 Adaptive genetics

In identifying outlier loci or adaptive loci, we sought to determine how selection may play a role in shaping genetic differentiation and adaptation along sharp environmental clines. All 42 outlier loci identified by both BayeScan and Dfdist were undergoing putative diversifying selection and balancing selection (Figure 4). Most of the outlier associated with environmental predictors across the alpine environmental gradient (Table 4), suggesting these regions of the genome seem to be diverging and that climate may play a role. Most outliers were associated with temperatures (especially BIO1 and BIO3), probably due to the steep gradient in temperatures along our sampled region. In addition, many outliers were associated with precipitation and landform of mountain, suggesting that precipitation and landform may also be exerting spatially divergent pressure on genetic. As expected, temperature and precipitation were estimated as the major driving factors influencing allele frequencies at outlier loci, consistent with other studies examining drivers of adaptive genetic divergence in plants (Manel et al., 2010; Yoder et al., 2014). Temperatures and precipitation factors are very important for plant growth, development, survival, reproduction, and defense (Poncet et al., 2010).

Previous studies have shown that the effect of habitat heterogeneity on diversification of *Rhododendron* species was stronger (Shrestha et al., 2018). In this study, we also found many outlier loci were related to the relief factors, such as 5 outlier loci were related to topographic position index (tpi), 4 outlier loci were related to aspect (asp), and 2 outlier loci were related to slope (slp) with high values of $R_{\text{adj}}^{2}$. The relief has complex indirect effects on the combination of snow distribution and slope‐specific interception of radiation and has the direct influence of exposure on microclimate during the plant growing season (Körner, 2003). In fact, changes in TPI, ASP, and SLP are responsible for the difference in habitat of *R. aureum*.

### 4.3 Distribution of *R. aureum*

We used MAXENT to predict the distribution of *R. aureum* under last glacial maximum (LGM), present, and future climate conditions. MAXENT captured well a major portion of current distribution of *R. aureum*. With the climate changing from the LGM to future, *R. aureum* decreased its future distribution range under a climatic warming scenario, especially under the Representative Concentration Pathways (RCP) 85 scenario which higher level greenhouse gases are emitted than RCP 26 in the years to come.

Previous studies indicated that *Rhododendron* species generally would be negatively affected by the climatic and land‐use change, and some distribution areas of narrow‐ranging *Rhododendron* species would decrease or even go extinct (Yu et al., 2019). We also found the suitable distribution range of *R. aureum* would be reduced to the high altitude tundra area but would lose the low altitude area in Changbai Mountain. In addition, Northeast Asia is the area of origin *Rhododendron*, which mostly prefer the cool climate of high latitudes (Shrestha et al., 2018). Therefore, with the warming of the climate, *R. aureum* may also appear the trend of migration to higher latitudes. This is consistent with previous studies on other alpine areas.

Climate change is causing many species to shift their geographical ranges as reviewed in many researches (Bellard et al., 2012; Dawson et al., 2011). The abundance and dominance of shrub species have increased in alpine and subarctic tundra ecosystems in recent decades (Brandt et al., 2013; Myers‐Smith et al., 2011, 2015), and climate warming has been considered the dominant factor driving these range expansions of shrubs (Brandt et al., 2013; Li et al., 2016; Naito & Cairns, 2011; Yu et al., 2010). Our results also suggest that alpine tundra will become a concentrated distribution area of *R. aureum* in future. However, climate‐induced range shifts and population declines are expected to increase the prevalence of population bottlenecks and reduce genetic diversity within and among species. Long‐lived species are particularly vulnerable to climate changes because they experience longer generation times, lower population turnover rates, and slower rates of evolution (Staudinger et al., 2012). In future, it is likely that the genetic diversity of *R. aureum* will decrease and thus affect its survival.

## 5 CONCLUSIONS

In summary, by using AFLP markers, landscape genetic, and species distribution modeling analysis together, we are able to identify many environmental factors that have influenced the genetic diversity and genetic structure, and we can predict the potential distribution area of *R. aureum*. Our analyses revealed high genetic variation and differentiation among populations and moderate levels of genetic diversity within populations of *R. aureum*. A significant correlation between genetic distance and environmental distance was identified, which suggested that environmental factors were the primary cause of the population differentiation. 42 outlier loci were identified in 36 populations of *R. aureum* alone the environmental gradient, and most of the outlier loci are associated with environmental factors, suggesting that these loci are linked to genes that are involved in the adaptability of *R. aureum* to environment. The SDM indicates that climate change drastically reduces the potential distribution range of *R. aureum*. An urgent area of future study is identification of genomic regions that are associated with environment factors by RAD‐Seq (Hohenlohe et al., 2012) and EST (expressed sequence tags). We should take measures to protect this species, such as translocate the populations or establish captive populations that would otherwise go extinct.

## CONFLICT OF INTEREST

None declared.

## AUTHOR CONTRIBUTIONS

**Wei Zhao:** Data curation (equal); formal analysis (equal); methodology (equal); writing‐original draft (lead). **Xiaolong Wang:** Formal analysis (equal). **Lin Li:** Methodology (equal). **Jiangnan Li:** Data curation (equal). **Hang Yin:** Methodology (equal). **Ying Zhao:** Methodology (equal). **Xia Chen:** Conceptualization (equal); writing‐review & editing (equal).

## Supporting information

Appendix S1

## Data Availability

The dataset that associated with this study is available at DRYAD (datadryad.org) with https://doi.org/10.5061/dryad.vmcvdncsn.
