# Supplementary material for: Evaluation of environmental factors affecting the genetic diversity, genetic structure, and the potential distribution of Rhododendron aureum Georgi under changing climate
Source: Ecol Evol. 2021 Aug 25;11(18):12294–306. doi: 10.1002/ece3.7803 (PMC8462154; doi:10.1002/ece3.7803)
Supplement: Supplementary file 1 — Appendix S1 [file ECE3-11-12294-s001.docx]

Appendix A

**Table A.** The primers used for AFLP analysis

| **Adaptors** | | |
| --- | --- | --- |
|  | EcoRI-1 5’-CTCGTAGACTGCGTACC-3’ | MseI-1 5’-GACGATGAGTCCTGAG-3’ |
|  | EcoRI2 5’-AATTGGTACGCAGTCTAC-3’ | MseI-2 5’-TACTCAGGACTCAT-3’ |
| **Preamplification primers** | | |
|  | 5’-GACTGCGTACCAATTCA-3’ | 5’-GATGAGTCCTGAGTAAC-3’ |
| **Selective amplification primers** | | |
| AFLP-1 | 5’-GACTGCGTACCAATTCACC-3’ | 5’-GATGAGTCCTGAGTAACAA-3’ |
| AFLP-2 | 5’-GACTGCGTACCAATTCACC-3’ | 5’-GATGAGTCCTGAGTAACTA-3’ |
| AFLP-3 | 5’-GACTGCGTACCAATTCAGG-3’ | 5’-GATGAGTCCTGAGTAACAT-3’ |
| AFLP-4 | 5’-GACTGCGTACCAATTCAGG-3’ | 5’-GATGAGTCCTGAGTAACAG-3’ |
| AFLP-5 | 5’-GACTGCGTACCAATTCACA-3’ | 5’-GATGAGTCCTGAGTAACTA-3’ |
| AFLP-6 | 5’-GACTGCGTACCAATTCACA-3’ | 5’-GATGAGTCCTGAGTAACTT-3’ |
| AFLP-7 | 5’-GACTGCGTACCAATTCACT-3’ | 5’-GATGAGTCCTGAGTAACAA-3’ |
| AFLP-8 | 5’-GACTGCGTACCAATTCACT-3’ | 5’-GATGAGTCCTGAGTAACAT-3’ |
| AFLP-9 | 5’-GACTGCGTACCAATTCACT-3’ | 5’-GATGAGTCCTGAGTAACAC-3’ |
| AFLP-10 | 5’-GACTGCGTACCAATTCACT-3’ | 5’-GATGAGTCCTGAGTAACTA-3’ |

Appendix B

Table B. The distribution records of *R. aureum*

| Sites | Longitude/E | Latitude/N | Sites | Longitude/E | Latitude/N |
| --- | --- | --- | --- | --- | --- |
| 1 | 137.8000 | 35.80000 | 22 | 128.0485 | 42.03802 |
| 2 | 142.8667 | 43.71667 | 23 | 128.0385 | 42.02957 |
| 3 | 142.8833 | 43.55000 | 24 | 128.0515 | 42.04300 |
| 4 | 142.8667 | 43.55000 | 25 | 128.0001 | 41.99007 |
| 5 | 142.8667 | 43.68333 | 26 | 128.0119 | 41.98912 |
| 6 | 138.3667 | 36.00000 | 27 | 128.0049 | 41.98713 |
| 7 | 128.0664 | 42.02920 | 28 | 128.0006 | 41.97795 |
| 8 | 128.0678 | 42.04018 | 29 | 128.0636 | 41.97879 |
| 9 | 128.0686 | 42.04202 | 30 | 128.0826 | 41.95675 |
| 10 | 128.0706 | 42.04575 | 31 | 128.0794 | 41.95501 |
| 11 | 128.0699 | 42.05552 | 32 | 128.0762 | 41.95376 |
| 12 | 128.0630 | 42.05935 | 33 | 128.0752 | 41.95178 |
| 13 | 128.0687 | 42.09014 | 34 | 128.0488 | 41.94115 |
| 14 | 128.1910 | 42.13372 | 35 | 128.0714 | 42.05420 |
| 15 | 128.0250 | 41.99727 | 36 | 128.0738 | 42.04927 |
| 16 | 128.0249 | 42.00210 | 37 | 128.0749 | 42.04517 |
| 17 | 128.0212 | 42.01297 | 38 | 128.0681 | 42.04015 |
| 18 | 128.0252 | 42.01640 | 39 | 128.0431 | 44.10465 |
| 19 | 128.0425 | 42.02153 | 40 | 128.0426 | 44.10325 |
| 20 | 128.0402 | 42.02443 | 41 | 127.9020 | 41.72863 |
| 21 | 128.0433 | 42.03347 | 42 | 127.9011 | 41.72845 |

**Appendix C**

**
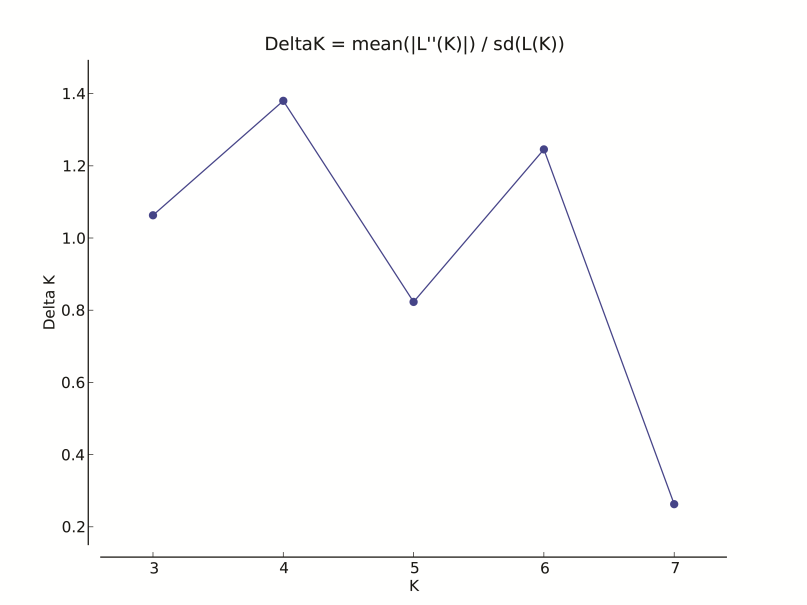
**

**Figure C. The modal value of this distribution is the true K, here 4 clusters.**

Appendix D


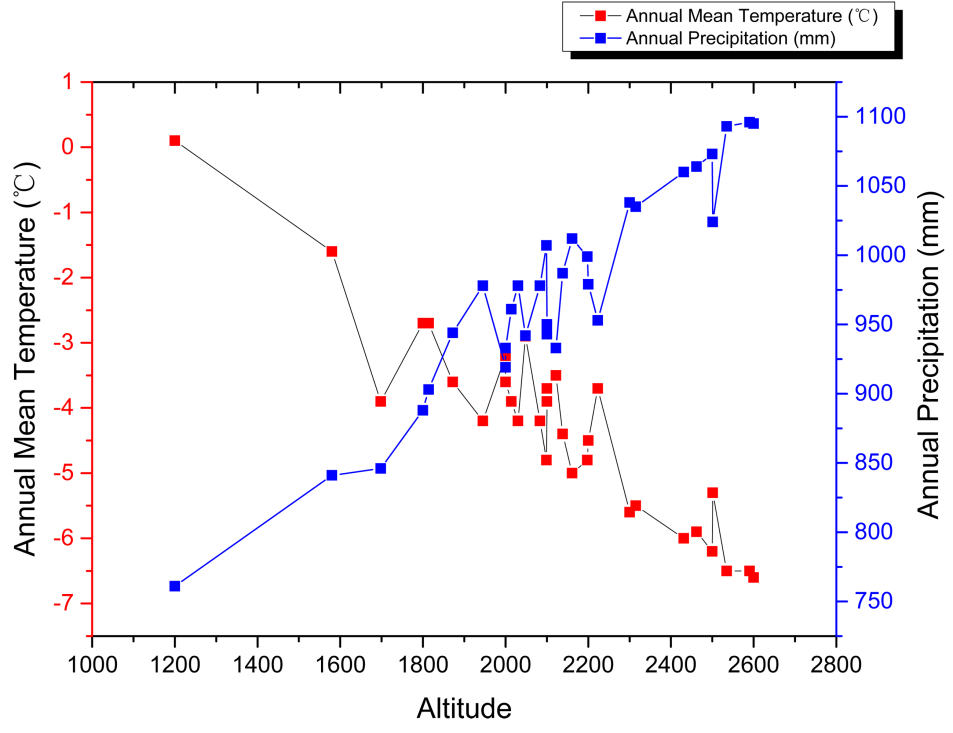


**Figure D.** The different annual mean temperature and annual precipitation along the altitude. The data was obtained from WordlClim database (<http://worldclim.org/>).
